# Removing the idiopathic from the chronic sensory neuropathies

**DOI:** 10.1093/brain/awab150

**Published:** 2021-05-13

**Authors:** Rhys C Roberts

**Affiliations:** Department of Neurology, Cambridge University Hospitals NHS Foundation Trust, Addenbrooke’s Hospital, Cambridge CB2 0QQ, UK

## Abstract

This scientific commentary refers to ‘*RFC1* expansions are a common cause of idiopathic sensory neuropathy’, by Currò *et al.* (doi:10.1093/brain/awab072).

**This scientific commentary refers to ‘*RFC1* expansions are a common cause of idiopathic sensory neuropathy’, by Currò *et al.* (doi:10.1093/brain/awab072).**

Uncertainty is a daily reality for a clinical neurologist. In response to reasonable requests for explanations about why particular symptoms and signs have emerged, how many times do we face our patients simply to reply, ‘I don’t know’? Of course, this would be a perfectly acceptable response and in keeping with our ethical obligations to be honest and trustworthy at all times; that is, doctors have always had to embrace the reality of ‘known unknowns’. Nevertheless, the medical lexicon has sometimes found ways of concealing gaps in knowledge with use of the terms ‘cryptogenic’ and ‘idiopathic’ directly linked to diagnostic labels,^1^ thereby conveying a sense of expertise and wisdom, with—in reality—little foundation, to the lay public. Removing terms such as ‘idiopathic’ and ‘cryptogenic’ from as many conditions as possible is therefore an important goal for all neurologists, made increasingly possible by the rapid advances in genome sequencing and processing, and this is exactly what Currò and colleagues^2^ have done for a significant subgroup of a commonly-seen and hitherto unexplained neurological presentation, as reported in this issue of *Brain*.

Peripheral neuropathies are common. Estimated to affect around 2.4% of the general population, rising to 8% in those over the age of 55,^3^ neuropathies can cause significant morbidity, and can sometimes be fatal. Peripheral nerves consist of motor nerves that convey motor signals from the spinal cord to the peripheries, sensory nerves that convey sensory signals from the peripheries to the CNS, along with sympathetic and parasympathetic nerve fibres that together make up the autonomic nervous system. While neuropathies can be classified into acute and chronic forms, they can also present symmetrically or in an asymmetric manner. Moreover, using neurophysiological and pathological criteria, neuropathies are often classified into demyelinating or axonal forms, reflecting the principal site of pathology as the myelin-forming Schwann cell or the axon, respectively. Furthermore, neuropathies can be classified into those that are inherited, such as the Charcot-Marie-Tooth and related disorders, and those believed to be acquired. Of the latter group, the commonest risk factor associated with an acquired peripheral neuropathy seen in European populations is diabetes mellitus, while infectious and toxic causes remain prevalent worldwide. Nevertheless, despite careful and thorough investigation, it is estimated that between 20% and 40% of neuropathies that present to the clinic have no identifiable cause—hence the longstanding and common use of ‘idiopathic’ or ‘cryptogenic’^4^—and are now grouped together under the diagnostic label of ‘chronic idiopathic axonal polyneuropathy’ (CIAP).^5^

The CIAPs mainly present with sensorimotor involvement (where both sensory and motor symptoms and signs exist), but a pure sensory neuropathy has also long been recognized, along with a rarer pure motor syndrome. Onset of symptoms is generally in the fifth decade and electrophysiological and histopathological studies are consistent with axonal degeneration. These neuropathies tend to deteriorate slowly and management is currently supportive and focused on symptoms. Previous observational studies aimed at identifying latent underlying risk factors and causes have been largely unsuccessful, hindering efforts to develop effective therapies.

In contrast, many other forms of neuropathy are treatable. While acquired inflammatory neuropathies are commonly managed with immunomodulation, the past decade has seen the development of novel yet effective therapies for previously untreatable inherited neuropathies.^6^^,^^7^ These treatments are the culmination of years of scientific investigation which began with the discovery of mutations in disease-associated genes in affected families, followed by the deciphering of molecular pathological mechanisms at the cellular level, leading finally to the identification of potential therapeutic pathways amenable to pharmacological targeting.

Twenty years after the publication of the first drafts of the human genome, our ability to sequence DNA has improved considerably, to the point where whole human genomes can now be sequenced in less than a day for less than £800. This has led to the identification of causative genetic changes that underpin many previously poorly understood neurological illnesses. Furthermore, while earlier discoveries related mostly to mutations in coding regions (exons), more recent work has unearthed a number of causal associations between neurological disorders and changes in non-coding regions of the genome. A recent but significant example is the identification of a biallelic intronic AAGGG repeat expansion in the replication factor C subunit 1 (*RFC1*) gene in patients presenting with CANVAS (cerebellar ataxia, neuropathy, vestibular areflexia syndrome) and also in patients presenting with late-onset ataxia.^8^^,^^9^ With an estimated carrier frequency of 0.7% in European populations, this genetic change is predicted to account for a significant proportion of patients presenting to neurologists. Furthermore, the likelihood of discovering the pathogenic pentanucleotide intronic repeat expansion increases significantly if the patient’s ataxia is associated with vestibular areflexia and a sensory neuropathy.

It is the prominence of the sensory neuropathy in CANVAS patients that led Currò and colleagues to investigate the possibility that the biallelic intronic *RFC1* AAGGG repeat expansion might account for a proportion of people given a diagnosis of sporadic CIAP. To this end, having identified a retrospective cohort of 225 patients from Italy and the UK with sensory (*n* = 125) or sensorimotor (*n* = 100) chronic axonal polyneuropathies without a family history, the authors went on to test for the presence of intronic *RFC1* repeat expansions in each case.

Revealingly, while no biallelic expansion was seen in any of the patients presenting with a mixed sensory and motor neuropathy (*n* = 0/100), biallelic *RFC1* intronic pathogenic expansions were detected in 34% (*n* = 43/125) of those with isolated sensory involvement. All but one of these *RFC1*-positive patients reported sensory symptoms at onset, which included numbness, paraesthesia and pain. In contrast, cerebellar or vestibular dysfunction at onset was rare. Intriguingly, and similar to the first reports of *RFC1*-positive CANVAS patients, a chronic dry cough was again a prominent symptom reported by 70% when asked directly, and was found to be a strong discriminating feature that could distinguish between sensory neuropathies harbouring the *RFC1* expansion and those that do not. Consistent with the earlier reports describing patients living with CIAPs, the median age of onset was 56 years (range 30–75) and the disease deteriorated slowly in the majority of cases (81%; *n* = 35/43), while otherwise being described as stable. Nevertheless, the observation that 10 years after symptom onset, over half the cohort displayed features of vestibular or cerebellar dysfunction (with 28% fulfilling the diagnostic criteria for CANVAS) suggests that *RFC1* disease forms a spectrum where a sensory neuropathy should be considered an early manifestation.

So what is the function of *RFC1*, and what is the underlying pathological mechanism? The *RFC1* gene, located on chromosome 4, encodes the largest of the five subunits that make up the ubiquitously-expressed human replication factor C (RFC) protein. This is known to be involved in DNA replication and repair, a cellular process already implicated in a number of autosomal recessive cerebellar ataxias associated with sensory neuropathies. However, the *RFC1* disease-associated intronic pentanucleotide repeat does not appear to alter the expression of the gene at the mRNA or protein levels, at least not in fibroblasts, lymphocytes, cortex or cerebellum. Furthermore, no obvious splicing abnormalities, expression of an antisense or non-coding transcript, or evidence of susceptibility to DNA damage have been found in the presence of the intronic repeat.^8^ Therefore, exactly how an intronic biallelic repeat expansion in the *RFC1* gene leads to a sensory neuropathy, later associated with vestibular and cerebellar dysfunction, remains unclear. Nevertheless, as a disorder that largely manifests in later life, it is likely that the molecular and cellular consequences of the intronic expansion will prove to be subtle and quite possibly beyond the threshold of current experimental detection. Moreover, what makes sensory peripheral neurons particularly susceptible to these potential deficits in DNA repair is a key question, the answer to which may point towards a common therapeutic target amenable to pharmacological manipulation.

Once a genetic change has been reported to be associated with a particular disorder, it is not uncommon for the phenotype of that disease (as clinically defined) to expand, which is an expected consequence of more widespread testing and awareness. This typically leads to incremental improvements in knowledge, for example the identification of new mutations in the same gene and the recognition of previously less-apparent features. By contrast, Currò and colleagues have instead recognized a clinical feature of the more complex neurological syndrome of CANVAS (i.e. the sensory neuropathy), and then asked whether the recently identified *RFC1* expansion might also account for the unexplained and previously unrelated sensory neuropathy seen in those diagnosed with CIAPs.

To this end, it must be highlighted that this approach was fully dependent on the ability of the investigators to retrospectively identify well-characterized cohorts of unrelated patients, the product of years of careful phenotyping in outpatient clinics, providing support for the notion that humans are indeed the ultimate animal model of human diseases.^10^ Furthermore, it is worth noting that over a quarter of the patients discovered to harbour the *RFC1* intronic expansion in this study had previously received an alternative and often-assumed acquired diagnosis. This emphasizes the need to always consider potential underlying genetic causes and factors when assessing patients who present with subacute symptoms, even when no other affected family members are reported, and especially taking into account autosomal recessive inheritance and the phenomenon of incomplete penetrance.

Overall, Currò and colleagues have successfully and elegantly demonstrated the power of performing large-scale targeted genetic investigations in the era of widespread whole genome sequencing alongside careful and methodical clinical observations. In this way, the term idiopathic can now be permanently removed from a significant proportion of chronic sensory axonal neuropathies that present to neurology clinics. Nevertheless, while this might come as welcome news to neurologists who can now boast to knowing fewer ‘unknowns’ than before, we must quickly turn our attention to answering the next inevitable question that such studies will kindle: ‘So what can you do about it?’
